# Adolescents’ internalizing and externalizing symptom trajectories in relation to early-life parental depressive symptoms

**DOI:** 10.1192/j.eurpsy.2025.10067

**Published:** 2025-07-14

**Authors:** Zsófia Csajbók, Pavla Brennan Kearns

**Affiliations:** 1 Faculty of Humanities, Charles University, Prague, Czech Republic; 2 Second Faculty of Medicine, Charles University, Prague, Czech Republic

**Keywords:** latent trajectories, mental health, parallel processes, relationship maintenance

## Abstract

**Background:**

While risk factors for children’s internalizing and externalizing symptom trajectories have been widely studied, their association with parental depressive symptom trajectories has yet to be explored.

**Methods:**

We used data from a prospective birth cohort of 2,542 Czech children and their parents. Children reported internalizing and externalizing symptoms at ages 11, 15, and 18 years. Parental depressive symptoms were assessed eight times from the prenatal period to the child’s age of 11 years. Latent Class Growth Mixture Modeling identified parallel trajectories of children’s symptoms. Five parental depressive symptom trajectories were adopted from previous research.

**Results:**

We identified four distinct classes of children’s symptom trajectories: (1) low internalizing and low externalizing (64%), (2) low internalizing and high externalizing (8%), (3) elevated internalizing and elevated externalizing (19%), and (4) high internalizing and elevated externalizing symptoms (9%). Children were more likely to experience any symptoms if their mothers had elevated depressive symptoms. High maternal and paternal depressive symptoms were associated with high internalizing and elevated externalizing symptoms in children. Constantly depressed mothers with elevated depressive symptoms in fathers had a high likelihood of any symptom trajectories in children. Other strong predictors of children’s symptom trajectories included parental relationship status (e.g., divorce), prior abortion, as well as children’s sex, urban versus rural residence, stressful life events, and self-esteem.

**Conclusions:**

Parents’ and children’s mental health trajectories are interconnected. Given the strong influence of parental relationship dynamics on both parental and child mental health, interventions should prioritize mitigating relationship strains to support family well-being.

## Introduction

Adolescent and young adult mental health is an increasing concern, as recent studies indicate worsening of both internalizing and externalizing symptoms [1, 2]. Internalizing symptoms encompass anxiety, sadness, social withdrawal, and challenges in peer interactions, while externalizing symptoms involve hyperactivity, impulsivity, and behavioral difficulties. The state-of-the-art approach for tracking mental health trends in young people relies on identifying patterns in long-term data, where subgroups exhibit distinct longitudinal symptom trajectories [3–10]. Because internalizing and externalizing symptoms often develop in parallel within the same child, analyzing their joint trajectories offers a more comprehensive and data-driven perspective than examining each symptom type separately.

A comprehensive understanding of adolescent mental health requires examining family dynamics as a whole, as families function as interconnected units [11]. It is well established that parental depressive symptoms can contribute to the worsening of both internalizing and externalizing symptoms in children [12, 13]. In our previous study [14], we applied a dyadic, parallel-process approach to analyze parental depression trajectories in a data-driven manner. We identified five distinct classes of maternal and paternal depression trajectories spanning from before the child’s birth until age 11, with symptom levels remaining stable over time. The most common trajectory (42%) included couples in which both parents exhibited consistently low depressive symptoms. This was followed by a group in which the mother had elevated depressive symptoms while the father’s remained low (24%), and another, in which both parents exhibited elevated depressive symptoms (20%). Less frequent patterns included couples where the mother was consistently depressed while the father had elevated symptoms (9%), and those where both parents remained chronically depressed (5%).

Several studies have examined trajectories of parental depressive symptoms [13, 15–20] as well as children’s internalizing and externalizing symptoms [3–10]. Yet, to the best of our knowledge, no prior research has explored the association between parental depression trajectories and the joint development of offspring symptom trajectories. Understanding these familial patterns can help practitioners identify at-risk families and intervene effectively.

Beyond parental depressive symptoms, numerous other factors – such as parental health, relationship dynamics and quality, and household socioeconomic status – may influence the development of internalizing and externalizing symptoms in children [3–10]. Leveraging a well-characterized birth cohort with follow-up into young adulthood, our study aimed to: (1) identify trajectories of internalizing and externalizing symptoms in young people as parallel processes, (2) examine their association with parental depressive symptom trajectories, and (3) explore additional family-related factors that may contribute to these developmental patterns.

## Methods

### Participants and procedure

We analyzed data from the Czech arm of the European Longitudinal Study of Pregnancy and Childhood (ELSPAC-CZ) [21], a prenatal cohort tracking children born between 1991 and 1992 in two Czech towns, Brno and Znojmo. Mothers were recruited during the second or third trimester of pregnancy, and both parents completed multiple questionnaires about themselves and their child until the child’s adulthood. For this study, we used parental reports collected during the prenatal and newborn periods, as well as at child ages 6 months, 18 months, 3 years, 5 years, 7 years, and 11 years. Children provided self-reported data at ages 11, 15, 18, and 19 years. The final sample consisted of 2,542 families, who had at least one measure of parental depressive symptoms and offspring internalizing or externalizing symptoms. All participants gave written informed consent, and the study received ethical approval from the ELSPAC-CZ Ethics Committee.

### Measures

#### Internalizing and externalizing symptoms of children

The Strengths and Difficulties Questionnaire (SDQ) [22] was administered to children at ages 11, 15, and 18 years to assess their behavioral and emotional difficulties. The SDQ consists of five subscales: emotional symptoms, conduct problems, hyperactivity/inattention, peer relationship problems, and prosocial behavior. The internalizing symptoms score was calculated by summing the responses from the *emotional symptoms* and *peer relationship problems* subscales. Higher scores reflect greater difficulties in anxiety, sadness, social withdrawal, and peer interactions. The externalizing symptoms score was computed by summing the responses from the *conduct problems* and *hyperactivity/inattention* subscales. Higher scores indicate behavioral regulation issues, impulsivity, aggression, and rule-breaking tendencies. If responses to individual SDQ items were missing, a prorated mean approach was applied when no more than two items per subscale were missing. Then, the internalizing and externalizing symptoms scores were created only for those people who have all the subscales. Each item in the SDQ at ages 15 and 18 years was rated on a three-point Likert scale (*0 = not true, 1 = somewhat true, 2 = certainly true*), but at 11 years, each item was rated on a four-point Likert scale (*0 = not true, 1 = seldom true, 2 = often true, 3 = always true*). Therefore, we divided the internalizing and externalizing symptoms scores by four at age 11 and by three at ages 15 and 18 to bring them into the same measurement scale.

#### Depressive symptoms of parents

Parental depressive symptoms were assessed using the Edinburgh Postnatal Depression Scale (EPDS) [23], a widely used self-report questionnaire designed to screen for depressive symptoms in parents. It was administered to the mothers and fathers in the prenatal and newborn period, and at 6 months, 18 months, 3 years, 5 years, 7 years, and 11 years of age of the child. The EPDS consists of 10 items that evaluate emotional and psychological well-being over the past 7 days. Each of the 10 items is rated on a four-point Likert scale (0 to 3), with total scores ranging from 0 to 30. If three or less items were missing, the arithmetic mean of the other items replaced the missing item. In case four or more items were missing, the variable was considered missing. Higher scores indicate more depressive symptoms, including feelings of sadness, anxiety, guilt, and suicidal ideation.

#### Other characteristics

Children self-reported information on their well-being (satisfaction with life [24], stressful life events, self-esteem [25]). Parents provided data on their demographic data (age, education, town), socioeconomic situation (income, crowding ratio, deprivation, employment, financial help, living in own house, amenities, utilities, social network, social support, childcare situation), health (number of diseases, substance use, smoking, alcohol use), mother’s obstetric history (number of previous pregnancies, own children, miscarriages, abortions, obstetric treatment), their family history (stressful life events, parental care, overprotection, home stability, sexual abuse), relationship maintenance, emotional life (affection, aggression, love of the baby, newborn temperament). Detailed information about the covariates can be found in the Supplementary Material.

### Data analysis

Descriptive statistics of internalizing and externalizing symptoms scales with Cronbach’s alphas (.66–.76) are presented in Supplementary Table S1. First, we extracted longitudinal internalizing and externalizing symptoms with parallel processes Latent Class Growth Mixture modeling (see details in the Supplementary Material). This method identifies latent classes in the sample that follow similar longitudinal patterns. The parallel processes specification considers the co-evolution of the two symptom types (i.e., the joint patterns) instead of identifying internalizing or externalizing symptom trajectories independently from each other. Based on selected model criteria, we chose the 4-class model for reporting with caveats, because the entropy (i.e., the differentiating ability between the classes) was relatively low (.52; Supplementary Tables S2–S5).

Second, we contrasted the four classes of offspring internalizing and externalizing symptoms with five classes of parental depressive symptom trajectories (see analysis described elsewhere [14]: Class P1: Mother has elevated depression, father is non-depressed (M elevated-F low); Class P2: both mother and father have elevated depression (M elevated-F elevated); Class P3: both mother and father are non-depressed (M low-F low); Class P4: both mother and father are depressed (M high-F high); and Class P5: mother is constantly depressed, father has elevated depression (M high-F elevated). That is, parents of all children in this dataset were identified as having one of five longitudinal patterns of depressive symptoms and were compared with offspring symptom trajectories. We used a χ^2^ test and multinomial regressions, estimating odds ratios (OR) with 95% confidence intervals for the association between the five classes of parental depressive symptoms (using as reference category the Class P3 M low-F low) with the four classes of offspring internalizing and externalizing symptoms (using as reference category the Class O1 with low internalizing and low externalizing symptoms). Third, we compared groups of continuous covariates across the four offspring symptom trajectories with ANOVA if the homogeneity of variances applied or the Brown-Forsythe test if it did not. The *post hoc* group comparisons were done with Hochberg multiple comparison tests. Binary covariates across the four classes were tested with χ^2^ tests. In the main text, we presented results of a certain group of covariates if any variable in that category group had effect sizes larger than *η* ^2^ > .01 or Cramer’s *V* > .05.

## Results

**Figure 1.**
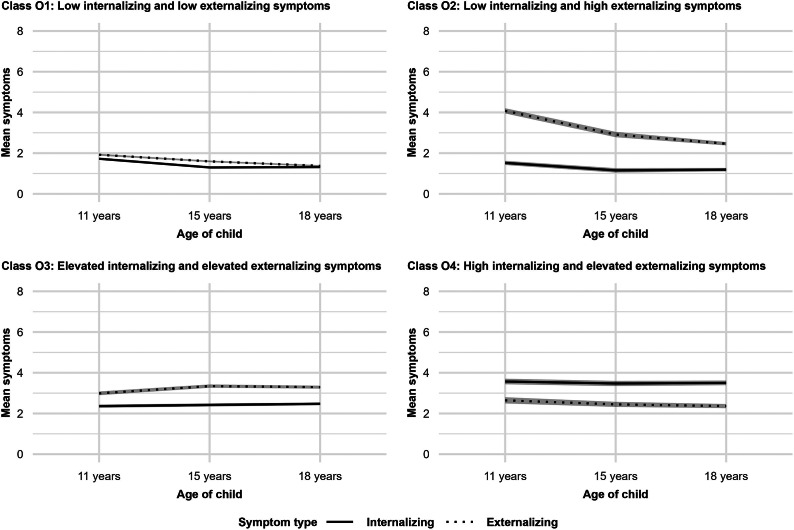
Offspring mean internalizing and externalizing symptoms in the four latent classes at 11, 15, and 18 years of age (95% confidence intervals shaded in gray).

Raw internalizing and externalizing symptoms scores weakly correlated with maternal and paternal depressive symptom scores at all available measurement points (Supplementary Table S6). The four identified offspring internalizing and externalizing symptom trajectories were Class O1: Low internalizing and low externalizing symptoms (INT low-EXT low, 64.08%); Class O2: Low internalizing and high externalizing symptoms (INT low-EXT high, 7.87%); Class O3: Elevated internalizing and elevated externalizing symptoms (INT elevated-EXT elevated, 18.96%); and Class O4: High internalizing and elevated externalizing symptoms (INT high-EXT elevated, 9.09%; Figure 1 and Table 1). Both symptom types decreased slightly over time in Classes O1 (INT low-EXT low) and O2 (INT low-EXT high) and increased slightly in Class O3 (INT elevated-EXT elevated). The cross-tabulation between offspring and parental symptom trajectories is in Table 2 (χ^2^ [12] = 49.92, *p* < .001, *V* = .08). Multinomial regression controlled for offspring sex and parental age found that Class P1 (M elevated-F low) and Class P2 (M elevated-F elevated) posed greater risks for all offspring symptom trajectories (*n* = 2,035, Table 3). Class P4 (M high-F high) had the highest risk for children being in Class O4 (INT high-EXT elevated; OR = 2.87, *p* < .01). Class P5 (M high-F elevated) had a high risk for all offspring symptom trajectories. Unadjusted results on the full sample are in Supplementary Table S7.

**Table 1.** Class proportions and mean intercept and slope results in the 4-class model of offspring internalizing and externalizing symptom trajectories

	*N* of class members	% of total *N*	Mean internalizing latent intercept factor	Mean internalizing latent slope factor	Mean externalizing latent intercept factor	Mean externalizing latent slope factor
Class O1	1,629	64.08%	1.714***	−0.072***	1.945***	−0.073***
Class O2	200	7.87%	1.639***	−0.057***	3.625***	−0.187***
Class O3	482	18.96%	2.148***	0.032+	2.699***	0.084**
Class O4	231	9.09%	3.220***	0.017	2.517***	−0.025

*Note*: Variables in the analyses were divided by four at age 11 and by three at ages 15 and 18 to bring them into the same measurement scale (items were rated on a 0 to 3 scale at age 11 and on a 0 to 2 scale at ages 15 and 18). This means that the true intercepts and slopes yielded on the original scores would be higher than the intercepts and slopes received in this analysis.

+*p* < .10, **p < .01, *** *p* < .001.

**Table 2.** Frequencies of offspring internalizing and externalizing symptom trajectories (Classes O1–O4) across parental depressive symptom trajectories (Classes P1–P5)

		Offspring symptom trajectory classes				
Parental trajectory classes		Class O1: Low internalizing and low externalizing symptoms	Class O2: Low internalizing and high externalizing symptoms	Class O3: Elevated internalizing and elevated externalizing symptoms	Class O4: High internalizing and elevated externalizing symptoms	Total
Class P1: Mother has elevated depression, father is non-depressed	*n*	397	54	142	67	660
	% within offspring classes	24.4%	27.0%	29.5%	29.0%	26.0%
Class P2: Both mother and father have elevated depression	*n*	318	44	101	47	510
	% within offspring classes	19.5%	22.0%	21.0%	20.3%	20.1%
Class P3: Both mother and father are constantly non-depressed	*n*	735	70	161	73	1,039
	% within offspring classes	45.1%	35.0%	33.4%	31.6%	40.9%
Class P4: Both mother and father are constantly depressed	*n*	66	10	17	17	110
	% within offspring classes	4.1%	5.0%	3.5%	7.4%	4.3%
Class P5: Mother is constantly depressed, father has elevated depression	*n*	113	22	61	27	223
	% within offspring classes	6.9%	11.0%	12.7%	11.7%	8.8%
Total	*n*	1,629	200	482	231	2,542
	% within offspring classes	100.0%	100.0%	100.0%	100.0%	100.0%

**Table 3.** Multinomial regression predicting offspring internalizing and externalizing symptom trajectories (Classes O1–O4) with parental depressive symptom trajectories (Classes P1–P5) controlled for offspring sex and parental prenatal age (*n* = 2,035)

	Class O2: Low internalizing and high externalizing symptoms	Class O3: Elevated internalizing and elevated externalizing symptoms	Class O4: High internalizing and elevated externalizing symptoms
Offspring sex (female)	0.61** (0.44, 0.86)	1.28* (1.02, 1.61)	1.60** (1.17, 2.19)
Mother’s age	0.97 (0.92, 1.02)	1.01 (0.98, 1.05)	1.01 (0.96, 1.06)
Father’s age	1.01 (0.97, 1.05)	0.99 (0.97, 1.02)	0.99 (0.95, 1.03)
Class P1: Mother has elevated depression, father is non-depressed	1.55* (1.03, 2.33)	1.44* (1.08, 1.92)	1.66* (1.12, 2.46)
Class P2: Both mother and father have elevated depression	1.59* (1.02, 2.48)	1.36+ (0.99, 1.87)	1.76** (1.15, 2.68)
Class P4: Both mother and father are constantly depressed	1.32 (0.54, 3.23)	1.31 (0.71, 2.42)	2.87** (1.49, 5.55)
Class P5: Mother is constantly depressed, father has elevated depression	1.90* (1.03, 3.52)	2.42*** (1.62, 3.62)	2.46** (1.42, 4.28)

*Note*: The reference category in offspring symptom patterns is Class O1: Low internalizing and low externalizing symptoms, the reference category in parental symptom patterns is Class P3: Both mother and father are constantly non-depressed. Results are presented in odds-ratios.

+*p* < .10, *p < .05, **p < .01, *** *p* < .001.

We compared the offspring classes across parental relationship maintenance variables (Table 4). We found that parents of Class O1 children (INT low-EXT low) were the most likely to be continuously married, only married, and ever married. Parents of Classes O2 (INT low-EXT high) and O3 (INT elevated-EXT elevated) children were the most likely to divorce, and Class O1 (INT low-EXT low) were the least likely to. Classes O2 (INT low-EXT high) and O3 (INT elevated-EXT elevated) parents were the least likely to continuously cohabiting. When comparing them in demographic data, we found that Classes O3 (INT elevated-EXT elevated) and O4 (INT high-EXT elevated) were more likely female, and Class O2 (INT low-EXT high) was more likely male. Class O3 (INT elevated-EXT elevated) children were the most likely born in Brno, while Class O1 (INT low-EXT low) children were the least likely. Offspring mental health variables showed significant and articulated associations with the internalizing and externalizing symptom trajectory classes. At all ages, Classes O1 (INT low-EXT low) and O2 (INT low-EXT high) experienced less stressful life events than Classes O3 (INT elevated-EXT elevated) and O4 (INT high-EXT elevated). Classes O1 (INT low-EXT low) and O2 (INT low-EXT high) had higher self-esteem and life satisfaction than Classes O3 (INT elevated-EXT elevated) and O4 (INT high-EXT elevated).

**Table 4.** Comparison of the four internalizing and externalizing symptom trajectories across parental relationship maintenance, demographic data, and offspring mental health

	Class O1: Low internalizing and low externalizing symptoms	Class O2: Low internalizing and high externalizing symptoms	Class O3: Elevated internalizing and elevated externalizing symptoms	Class O4: High internalizing and elevated externalizing symptoms	*η* ^2^/*V*
Parental relationship maintenance					
Unstable relationship *n*, %	274 (16.8%)	40 (20.0%)	94 (19.5%)	36 (15.6%)	.04
Continuously married *n*, %	1,123 (68.9%)	127 (63.5%)	303 (62.9%)	150 (64.9%)	.06*
Only married *n*, %	1,129 (69.3%)	127 (63.5%)	303 (62.9%)	152 (65.8%)	.06*
Married ever *n*, %	1,552 (95.3%)	184 (92.0%)	446 (92.5%)	217 (93.9%)	.06*
Not first marriage *n*, %	270 (16.6%)	39 (19.5%)	86 (17.8%)	45 (19.5%)	.03
Married later *n*, %	89 (5.6%)	13 (6.8%)	20 (4.3%)	12 (5.2%)	.03
Bereavement *n*, %	35 (2.1%)	8 (4.0%)	16 (3.3%)	4 (1.7%)	.04
Separated *n*, %	85 (5.2%)	12 (6.0%)	37 (7.7%)	11 (4.8%)	.04
Divorced *n*, %	158 (9.7%)	33 (16.5%)	69 (14.3%)	26 (11.3%)	.07**
Single ever *n*, %	116 (7.1%)	17 (8.5%)	40 (8.3%)	23 (10.0%)	.03
Continuous cohabitation *n*, %	1,484 (91.1%)	169 (84.5%)	424 (88.1%)	212 (91.8%)	.07**
Demographic data					
Child’s sex (female) *n*, %	795 (48.8%)	71 (35.5%)	268 (55.6%)	137 (59.3%)	.11***
Town (Brno) *n*, %	1,359 (83.4%)	177 (88.5%)	450 (93.4%)	201 (87.0%)	.11***
Age (m.) (*M*, *SD*)	25.10 (4.68)	24.75 (4.36)	25.27 (5.01)	25.22 (4.97)	<.01
Age (f.) (*M*, *SD*)	28.13 (5.78)	27.94 (5.81)	28.24 (6.23)	28.25 (6.36)	<.01
Education (m.) (*M*, *SD*)	4.23 (1.99)	4.20 (1.96)	4.26 (1.97)	4.07 (1.86)	<.01
Education (f.) (*M*, *SD*)	4.21 (2.24)	3.94 (2.15)	4.11 (2.20)	4.04 (2.14)	<.01
Employed (f.) *n*, %	1,386 (93.9%)	171 (95.5%)	395 (91.6%)	189 (93.1%)	.04
Offspring mental health					
Stressful life events (11y) (*M*, *SD*)	0.40 (0.33)	0.47 (0.39)	0.52 (0.39)	0.61 (0.40)	.04***
Stressful life events (15y) (*M*, *SD*)	0.34 (0.26)	0.44 (0.43)	0.59 (0.40)	0.58 (0.36)	.12***
Stressful life events (18y) (*M*, *SD*)	0.37 (0.24)	0.47 (0.27)	0.60 (0.31)	0.53 (0.26)	.12***
Stressful life events (19y) (*M*, *SD*)	0.28 (0.23)	0.43 (0.31)	0.44 (0.35)	0.45 (0.39)	.07***
Self-esteem (15y) (*M*, *SD*)	30.60 (4.05)	30.01 (4.49)	27.42 (4.22)	26.75 (4.93)	.12***
Self-esteem (18y) (*M*, *SD*)	31.96 (4.30)	30.65 (3.80)	28.21 (4.60)	27.44 (5.57)	.15***
Self-esteem (19y) (*M*, *SD*)	32.43 (5.12)	33.15 (3.44)	29.24 (5.82)	27.14 (5.18)	.12***
Satisfaction with life (15y) (*M*, *SD*)	18.36 (3.70)	17.57 (3.86)	16.27 (3.97)	15.86 (4.22)	.07***
Satisfaction with life (19y) (*M*, *SD*)	18.60 (4.23)	17.73 (4.34)	16.97 (4.62)	15.36 (4.76)	.06**

Abbreviations: m., mother; f., father; y, years of age.

*p < .05,**p < .01, ***p < .001.

When considering obstetric history, mothers of children in Classes O2 (INT low-EXT high) and O3 (INT elevated-EXT elevated) experienced the most likely abortion, while mothers of children in Classes O1 (INT low-EXT low) and O4 (INT high-EXT elevated) experienced abortion the least likely (Table 5). Mothers of Classes O2 (INT low-EXT high) and O3 (INT elevated-EXT elevated) children had a higher prevalence of smoking than mothers of Classes O1 (INT low-EXT low) and O4 (INT high-EXT elevated). Fathers of Class O1 (INT low-EXT high) children had a lower prevalence of current smoking status than in the other classes. When comparing newborn temperament, parental emotional life, socioeconomic resources, parental childhood history, and parental depressive symptom scores across the four classes, we found no or negligible differences (Supplementary Tables S8–S10). To evaluate misclassification bias from low entropy, we performed sensitivity analyses on participants with class membership certainty over 50% (*n* = 2,128). Overall, potential misclassification did not significantly affect covariate comparisons, but class differences in continuous cohabitation and smoking status disappeared (see details in the Supplementary Tables S11–S13).

**Table 5.** Comparison of the four internalizing and externalizing symptom trajectories across obstetric history and parental health

	Class O1: Low internalizing and low externalizing symptoms	Class O2: Low internalizing and high externalizing symptoms	Class O3: Elevated internalizing and elevated externalizing symptoms	Class O4: High internalizing and elevated externalizing symptoms	*η* ^2^/*V*
Obstetric history					
Previous pregnancy *n*, %	823 (63.1%)	111 (66.9%)	250 (63.8%)	117 (61.6%)	.02
First pregnancy with partner *n*, %	159 (19.8%)	29 (26.6%)	63 (25.9%)	25 (21.7%)	.07
Miscarriage *n*, %	203 (24.7%)	30 (27.3%)	58 (23.3%)	38 (32.5%)	.06
Abortion *n*, %	272 (33.1%)	43 (38.7%)	112 (44.8%)	39 (33.6%)	.10**
Birth of dead child ever *n*, %	29 (3.5%)	4 (3.6%)	9 (3.6%)	7 (6.0%)	.04
Obs. treatment *n*, %	45 (36.6%)	8 (42.1%)	14 (33.3%)	12 (35.3%)	.05
*N* of previous pregnancies (*M*, *SD*)	1.74 (1.05)	1.82 (1.41)	1.93 (1.17)	1.97 (1.58)	.01
*N* of children (*M*, *SD*)	1.00 (0.69)	0.95 (0.74)	1.07 (0.76)	1.04 (0.97)	<.01
*N* of miscarriage (*M*, *SD*)	1.17 (0.49)	1.14 (0.35)	1.18 (0.43)	1.30 (0.70)	.01
*N* of abortion (*M*, *SD*)	1.29 (0.60)	1.30 (0.67)	1.30 (0.58)	1.41 (0.85)	<.01
Parental health					
Smoking (m.) *n*, %	172 (12.2%)	29 (16.1%)	74 (17.5%)	22 (10.7%)	.07*
Smoking (f.) *n*, %	464 (36.9%)	74 (46.0%)	171 (45.7%)	80 (43.0%)	.08**
Substance use (m.) *n*, %	17 (1.3%)	1 (0.6%)	6 (1.5%)	6 (3.1%)	.05
Substance use (f.) *n*, %	46 (2.9%)	8 (4.2%)	14 (3.0%)	9 (4.1%)	.03
*N* of diseases (m.) (*M*, *SD*)	0.69 (1.00)	0.67 (1.06)	0.94 (1.17)	0.95 (1.19)	.01***
*N* of diseases (f.) (*M*, *SD*)	0.70 (1.05)	0.82 (1.20)	0.76 (1.27)	0.84 (1.12)	<.01
Alcohol use (m.) (*M*, *SD*)	0.15 (0.64)	0.15 (0.57)	0.15 (0.66)	0.22 (0.86)	<.01
Alcohol use (f.) (*M*, *SD*)	1.61 (1.31)	1.76 (1.44)	1.69 (1.39)	1.61 (1.41)	<.01
Psychotropic pills (m.) (*M*, *SD*)	1.04 (0.23)	1.10 (0.36)	1.08 (0.35)	1.08 (0.33)	.01*
Psychotropic pills (f.) (*M*, *SD*)	1.04 (0.24)	1.06 (0.25)	1.04 (0.21)	1.07 (0.35)	<.01

Abbreviations: m., mother; f., father.

* *p* < .05, **p < .01, ***p < .001.

## Discussion

We identified four classes of longitudinal internalizing and externalizing symptom trajectories over the whole of adolescence. The most common group (64%) was adolescents who had both internalizing and externalizing symptoms low and decreasing, followed by a group of both internalizing and externalizing symptoms elevated and increasing, particularly externalizing symptoms (19%). Less common were adolescents with high internalizing and elevated externalizing symptoms (9%), and low internalizing and high externalizing symptoms, but both were decreasing (8%). We contrasted children’s symptom trajectories with parents’ depressive symptoms trajectories prior to children’s adolescence. We found that 1) elevated maternal depressive symptom trajectories – coupled with either no paternal depression or with elevated paternal depression – were associated with increased likelihood for children being in any of the elevated or high symptom trajectory groups; 2) having any of the elevated or high internalizing or externalizing symptoms in children was associated with high maternal and elevated paternal depressive symptom trajectories; 3) both constantly depressed parents most likely had children who had high internalizing and elevated externalizing symptoms. Children’s symptom trajectory patterns were strongly associated with parental relationship maintenance, their own stressful life events, life satisfaction, self-esteem, demographic data, mother’s obstetric history, and maybe parental smoking. On the contrary, children’s trajectories of internalizing and externalizing symptoms were not strongly associated with newborn temperament, parental emotional life, socioeconomic resources, or parental childhood history.

Comparing joint trajectories of internalizing and externalizing symptoms across studies is challenging due to differences in sampling, age periods observed, and thresholds for symptom classification. Previous research identified three to five trajectory classes [3–10]. In our study, the largest group – over 60% of the sample – had low internalizing and externalizing symptoms. Other studies found an even larger low-symptom group: over 70% [8, 10] and more than 80% [4, 5]. Conversely, some studies reported a smaller low-symptom group, with less than 50% [6], about 30% [3, 7], and under 30% [9]. Patterns of comorbid symptoms also varied. Like two other studies [7, 10], we did not identify a high-risk group with both high internalizing and externalizing symptoms. However, other studies reported joint high or very high symptoms in 3% [8], 4% [5], 6% [4], 10% [6], 27% [3], and 30% [9] of participants.

Several correlates of trajectories from previous studies align with our findings. Previous studies found that riskier trajectories of internalizing and externalizing symptoms are associated with various factors, including parental marital problems, low family income, an absent father, maternal mental health issues, male sex, parental tobacco use, other substance use, speech difficulties, and the child’s temper tantrums [3]. Additionally, childhood trauma, maltreatment, and suicidal ideation [4], poor decision-making, being a bully or a bullying victim, engaging in antisocial behaviors, skipping and disliking school, low self-esteem, and overall unhappiness [5] were linked to these trajectories. Other related factors include respiratory sinus arrhythmia [6], difficulties transitioning into adulthood [7], substance use and problematic behavior [8], an ego-resilient personality, higher teacher–child relationship conflict, and being an ethnical minority [9]. Low language ability and peer rejection were identified as unique antecedents for the chronic co-occurring group [9], while harsh parenting was also a contributing factor [10].

More specifically, the parental depressive pattern most strongly associated with children’s symptom trajectories involved a consistently depressed mother and a father with elevated depressive symptoms. This pattern more than doubled the odds of children following any of the three trajectories: low internalizing with high externalizing symptoms (though this association disappeared in the sensitivity analysis, possibly due to lower power); elevated internalizing with elevated externalizing symptoms; and high internalizing with elevated externalizing symptoms. Surprisingly, persistent depression in both parents was only associated with high internalizing and elevated externalizing symptoms, highlighting the importance of balanced (i.e., similar) parental mental health for child outcomes. Shared depressive symptoms may foster greater intimacy between parents, potentially improving functioning in some areas [26]. It is also possible that, in these cases, other mechanisms – such as genetic factors – underlie the heightened risk of internalizing symptoms [27].

Similarly, when both parents had elevated depression, there was a moderate increase in the likelihood of children experiencing elevated internalizing and externalizing symptoms. However, when only the mother had elevated depression and the father did not, the risk was even higher – particularly in the sensitivity analysis. This reinforces that disparities in parental mental health – particularly when the mother is worse off – can be especially detrimental to children’s well-being. It is well known that maternal depressive symptoms are associated with internalizing problems in children [28]. As primary caregivers, mothers typically provide crucial emotional support, and their mental health strongly influences children’s emotional regulation. A more severely depressed mother than father may be especially harmful by disrupting caregiving stability, increasing family conflict, and placing emotional burden on children. Thus, when the father is less affected, the burden of compensating for his partner’s depression may strain him, in turn contributing to greater family tension and instability.

Further, marital status – one of the strongest correlates of parental depressive symptom trajectories [14] – was associated with children’s symptom trajectories found here as well. Consistent with other studies, children with lower longitudinal symptom levels were more likely to have parents who were married, consistently cohabiting (though this effect disappeared in the sensitivity analysis), and not divorced [29]. While prior work found parental depressive symptom trajectories more closely linked to separation [14], children’s symptom trajectories here were more strongly associated with parental divorce. Experiencing separation and divorce may manifest in many ways. Parents may be separated and get back together or may divorce without a prior separation period. We tentatively interpret separation – as a prolonged period of uncertainty regarding the relationship’s future – as the most stressful phase for parents. Children may remain hopeful during separation, or parents may not discuss it openly. In contrast, divorce is final and clear to children, thereby exerting a stronger impact on their mental health than separation.

Consistent with our study, Dutch children living in urban environments also exhibited more behavioral and emotional problems compared to those in rural areas [30]. A meta-analysis also found that psychiatric disorder rates are higher in urban than in rural areas [31]. Concerning our study, the population of Brno was more than 10 times greater than Znojmo’s during our participants’ formative years. Hence, it is plausible that children in Brno were exposed to more social (e.g., stress, isolation) and physical (e.g., air pollution, population density) stressors that posed a higher risk for mental health difficulties [31, 32]. Alternatively, better access to mental health services and greater social acceptance in urban settings may have caused reporting bias [33].

Similarly, previous studies found that children born from unintended pregnancies tend to have poorer bonding with their mothers compared to those from wanted pregnancies or pregnancies following abortion [34, 35]. In our study, mothers of children in the elevated internalizing and externalizing or low internalizing and high externalizing symptom groups were more likely to have had an abortion prior to the birth of the index child than mothers of children in the other two groups. To the best of our knowledge, no studies have directly compared the well-being of children born following abortion with those born from wanted pregnancies without prior abortion. However, families with children born after an abortion may continue to experience adversities that contributed to the abortion, including financial difficulties, poor maternal mental health, or substance use [36]. These adversities may have contributed to bonding difficulties and, ultimately, to mental health challenges in their offspring.

This study has several limitations. The internalizing and externalizing symptom scales had relatively low internal consistency, and the measurements at age 11 used different Likert scales than those at ages 15 and 18. As a result, our rescaling of the variables made it challenging to compare elevated and high symptom scores with findings from other studies. Additionally, the distinction between offspring trajectory classes was somewhat unclear (i.e., we had low entropy), which may have limited our ability to identify covariates associated with each symptom trajectory and influenced the prevalence estimates for each longitudinal symptom category. However, the performed sensitivity analyses indicated only a small bias stemming from misclassification. Participation rates at age 18 were notably low, and there was only one measurement point (at age 11) where both parental and offspring data were available. Future research could benefit from analyzing parallel trajectories of parental and offspring mental health while considering the family as a unit of measurement. Finally, since the cohort dates from the early 1990s Czechia, its findings may not generalize to modern cohorts or other countries, particularly as adolescent and young adult mental health problems have increased in recent decades [37].

Nonetheless, while prior research has linked parental depressive symptoms to increased internalizing and externalizing symptoms in children, our study advances the field by adopting a more nuanced family-level perspective. Rather than examining overall parent–child symptom associations, we explored how distinct family patterns shape adolescents’ outcomes. This pattern-specific approach acknowledges family heterogeneity and supports a more refined understanding of the mechanisms underlying adolescent mental health – pathways that may be obscured in generalized models.

To conclude, children’s internalizing and externalizing symptom trajectories were linked to parental depressive symptom trajectories. Elevated or high parental depression was linked to more child symptoms, especially when parents differed in symptom levels. Additionally, child’s sex, town of residence, stressful life events, self-esteem, and parental factors – such as relationship status and prior abortion before pregnancy – were among the strongest predictors of children’s symptom trajectories. Our findings offer nuanced insights into family mental health and underscore the importance of preventive interventions.

## Supporting information

10.1192/j.eurpsy.2025.10067.sm001 Csajbók and Brennan Kearns supplementary material Csajbók and Brennan Kearns supplementary material

## Data Availability

Access to the data is provided free of charge to researchers upon reasonable request. More information can be found on this website: elspac.cz. The study protocol and syntax of the statistical analysis will be shared upon request from the corresponding author of this study.

## Supplementary material

The supplementary material for this article can be found at http://doi.org/10.1192/j.eurpsy.2025.10067.
