# Supplementary material for: Adolescents’ internalizing and externalizing symptom trajectories in relation to early-life parental depressive symptoms
Source: Eur Psychiatry. 2025 Jul 14;68(1):e111. doi: 10.1192/j.eurpsy.2025.10067 (PMC12438980; doi:10.1192/j.eurpsy.2025.10067)
Supplement: Csajbók and Brennan Kearns supplementary material [file S0924933825100679sup001.docx]

**Adolescents’ internalizing and externalizing symptom trajectories in relation to early-life parental depressive symptoms**

Zsófia Csajbók^*^ & Pavla Brennan Kearns

*Corresponding author: Zsófia Csajbók, Ph.D., Faculty of Humanities, Charles University, Pátkova 5, Prague 8, 182 00, Czech Republic.

Email: [zsofia.csajbok@fhs.cuni.cz](mailto:zsofia.csajbok@fhs.cuni.cz)

**SUPPLEMENTARY MATERIAL**

**Supplementary Measures**

*Relationship maintenance* was categorized in multiple ways. Couples were assigned an “unstable relationship” status if they changed their relationship status at any point during the study. Those labeled as “only married” had no relationship status recorded other than their first marriage. Couples were considered “continuously married” if they had at least two confirmed reports of being married (first marriage) with no indication of separation, divorce, or bereavement. The “married ever” category included couples married at any point during the study, while “married later” applied to those who began the study single and married afterward. Additional codes included: “other than first marriage” (remarriage), “bereavement,” “separated,” “divorced,” “single” (at any point during the study), and “continuously cohabitated” for couples with no indication of living apart.

*Demographic* *data* included age of the parents, child’s sex (male, female), town (Brno, Znojmo), and education (1 = *primary* to 8 = *postgraduate education*). Data on *socioeconomic resources* concerned income (monthly household income), crowding (number of people living in the household / number of rooms in the household), deprivation (how difficult it is to secure basic things, such as food, clothes, rent, heating; higher values indicate greater deprivation), father’s employment (yes, no), financial help (if parents or other relatives help out financially; yes, no), living in own house (own house vs. with parents or other), amenities (sum of 10 household amenities, including fridge, washing machine, etc.), and utilities (own use of five basic utilities). We also included information about social network (sum of 10 items assessing the quality and the quantity of the relationships, where higher values indicate a greater network) and social support (sum of 10 items, where higher values indicate more social support). Further information was reported by the mother of the child at 6 months on childcare provided by other people (father, other family members, someone who is not family) expressed in hours per week and the age of the child (in months) when they started to take care of the child.

Information about *emotional life* concerned relationship aggression (how aggressive is the relationship with the partner, higher score indicates more aggression), affection (how affectionate is the relationship with the partner, higher score indicates more affection), and love of the baby (how long it took the mother to love the baby; immediately, shortly, over a week, don’t love them yet). Information about *health* concerned the number of diseases (sum of current 25 comorbidities), substance use (use of marijuana, psychostimulants, sleeping pills, crack, cocaine, or heroin; yes, no), smoking (non-smoker, current smoker), alcohol use (days in the past month consuming the equivalent of two beers, 0.5 l wine, four shots; 0 = *not one day* to 5 = *every day*), use of psychotropic pills (antidepressants, sedatives, or sleeping pills; 1 = *not at all* to 4 = *daily*). *Obstetric history* included data about previous pregnancy (yes, no), number of previous pregnancies (continuous), number of own children (continuous), history of miscarriage (yes, no), number of miscarriages (continuous), history of abortion (yes, no), number of abortions (continuous), and obstetric treatment (undergone any treatment for getting pregnant; yes, no).

Information about *parental history* includes stressful life events (sum of 41 events since the woman got pregnant), parental care (derived from items of the Parental Bonding Instrument, PBI, Parker et al., 1979, measuring how caring their mother was, with higher scores indicating more care), overprotection (derived from the PBI items measuring how overprotective their mother was, with higher scores indicating more overprotection), home stability (if their parents were predictable; 1 = *never*, 4 = *always*), and sexual abuse (if they experienced sexual abuse during childhood or adolescence; never, once, more than once).

*Offspring temperament* was assessed in the newborn questionnaire, in which parents rated 14 items on a Likert scale (1 = *not at all*, 4 = *very much*) based on how much the child shows the characteristics of being whiny, cranky, satisfied, etc. *Offspring mental health* was completed by the child and included internalizing and externalizing symptoms derived from the Strengths and Difficulties Questionnaire (where higher score indicates more symptoms, Goodman, 1997), satisfaction with life (a 5-item scale designed to measure global cognitive judgments of one’s life satisfaction, higher score indicates more satisfaction, Diener et al., 1985), and stressful life events (average of the ratings of experience of stressful events and how upset they made the respondent on a 0 = *it did not happen* to 4 = *it happened and made me very upset* scale; year 11: 20 events, year 15: 28 events, year 18 and 19: 33 events).

**Supplementary Data analysis**

*Identification of offspring internalizing and externalizing symptom trajectories*

Children’s internalizing and externalizing symptoms were measured at ages 11, 15, and 18 years (Table S1). To identify unique combined longitudinal internalizing and externalizing symptom trajectories, we defined a parallel processes model where children’s internalizing and externalizing symptoms were taken into account in parallel to each other. The analyses were carried out in Mplus 8.11. We considered having an excellent model fit if the root mean square error of approximation (RMSEA) and the standard root-mean-square residual (SRMR) model fit indices were lower than 0.08; and the comparative fit index (CFI) and Tucker-Lewis index (TLI) were higher than 0.90 (Brown, 2006). The parallel processes model with two parallel linear growth models specified (i.e., one for internalizing and another for externalizing symptoms) and correlated observed variables at each time point (i.e., correlated internalizing and externalizing symptoms at ages 11, 15, and 18 years each) had an excellent model fit on the overall sample (Table S2). Missing data was handled with the Full Information Maximum Likelihood Method.

Subsequently, we employed mixture modeling techniques that identify unique longitudinal trajectories among children’s parallel processes. First, we ran Growth Mixture Modeling that holds the variances of the latent growth factors equal across the identified latent classes (i.e., different subgroups of participants following similar trajectories). The analysis suggested that there are two latent groups in the data, however, the two identified patterns were qualitatively similar, that is, the groups only differed in the intercepts but not in the slopes (i.e., there was a group with high symptoms, 6.81%, and a group with low symptoms). Thus, we chose to run Latent Class Growth Mixture models, which constrain the variances of the latent growth factors to be zero.

Following the recommended guidelines, we relied on selected criteria in choosing the best model for reporting (Van De Schoot et al., 2017). These criteria tell us whether the extraction of k+1 classes can describe the data more meaningfully than the extraction of k classes. We started this iterative process with k=1, where the data is considered to be homogenous with no unique patterns emerging in them. The criteria we used were the Akaike Information Criterion (AIC; Akaike, 1973), Bayesian Information Criterion (BIC; Schwartz, 1978), entropy, the Vuong-Lo-Mendell-Rubin Likelihood Ratio Test (VLMR LRT; Vuong, 1989), the Lo-Mendell-Rubin Adjusted Likelihood ratio test (LMR ALRT; Lo et al, 2001), and the parametric bootstrapped likelihood ratio test (BLRT; McLachlan & Peel, 2004). The best model to stop at should have the lowest AIC and BIC among all of the tested k-class solutions with significant VLMRT LRT, LMR ALRT, and BLRT tests, and as high entropy as possible. Further, we considered interpretability and size of the smallest extracted class as indicators of model suitability.

We extracted 2-, 3-, 4-, and 5-class solutions and replicated them all with different numbers of random starts at least 3 times (Figures S1a-d). After that, we ran the LRT-starts option to obtain the parametric BLRT test. Based on the results, we selected the 4-class model to study, as the 5-class model yielded non-significant VLM RLR and LMR ALRT tests and small subsample sizes (Table S3). However, the 4-class solution has relatively low entropy, that is, the distinction between the classes is uncertain (Table S4). The model results of the 4-class LCGM solution can be found in Table S5. After that, we ran a sensitivity analysis on a subset of participants (*n* = 1459) who had at least 2 data points and on a subset of participants who had full data coverage (*n* = 469). We found that the patterns in general replicated very well across the total sample (*N* = 2542) and the subsample with better data coverage, but only partially with the subsample with full data coverage (Figures S2a-b).

Eventually, we performed an additional sensitivity analysis to test the influence of low entropy on the results. We filtered out participants, who had lower probability of their class memberships than 50% (*n* = 414). Class frequencies in the certain subsample (*n* = 2128) were Class O1 = 68.8%, Class O2 = 6.7%, Class O3 = 15.5%, and Class O4 = 9.1%. On the certain subsample of participants with more certain class memberships, we performed again the multinomial regression (Table S11), Brown-Forsythe analysis of variance, and χ^2^ tests (Tables S12 and S13) to compare with the results obtained on the overall sample (*N* = 2542, Tables 3­‑5).

We found a few notable differences. In the multinomial regression predicting offspring trajectories with parental depression trajectory classes, Class O2 was more strongly associated with Class P1 in the certain sample (OR = 1.87, *p* < .01) than in the overall sample (OR = 1.55, *p* < .05), and the associations declined and became non-significant with Class P2 (certain sample OR = 1.54, n.s., overall sample OR = 1.59, *p* < .05) and Class P5 (certain sample OR = 1.73, n.s., overall sample OR = 1.90, *p* < .05) in the certain sample in comparison to the overall sample. Also declined the association of Class O3 with Class P2 (certain sample OR = 1.10, n.s., overall sample OR = 1.36, *p* < .10) and the association of Class O4 with Class P5 (certain sample OR = 2.16, *p* < .05., overall sample OR = 2.46, *p* < .01).

Among the parental relationship maintenance variables, the associations weakened in the variables married ever (certain sample *V* = .05, *p* < .10, overall sample *V* = .06, *p* < .05) and continuous cohabitation (certain sample *V* = .05, n.s., overall sample *V* = .07, *p* < .01). The frequencies remained similar except that more parents lived together in Class O2 in the certain class membership (87.3%) subsample than in the overall sample (84.5%). Another difference was found in the association with town of residence. In the certain subsample, all the participants (100%) lived in Brno in Class O3 (*V* = .18, *p* < .001), while in the overall sample, only 93.4% (*V* = .11, *p* < .001). Finally, we found that the effect of smoking declined in both mothers (certain sample *V* = .04, n.s., overall sample *V* = .07, *p* < .05) and fathers (certain sample *V* = .06, n.s., overall sample *V* = .08, *p* < .05). These differences could be due to lower frequency of smoking mothers in Class O3 in the certain subsample (14.1%) than in the overall sample (17.5%) and lower frequency of smoking fathers in Class O2 in the certain subsample (37.8%) than in the overall sample (46.0%). The differences in significance between the overall sample and the certain subsample could be partially attributed to the lower sample size in the certain subsample. In general, the changes did not influence the interpretations significantly.

| Table S1. Descriptive statistics and data coverage of internalizing and externalizing symptom scores | | | | | | | | | |
| --- | --- | --- | --- | --- | --- | --- | --- | --- | --- |
| Strengths and Difficulties Questionnaire | | *N* | Cronbach’s alpha | Min. | Max. | Mean | *SD* | Skewness | Kurtosis |
| Internalizing | 11 years | 2325 | .66 | 0.00 | 6.58 | 2.00 | 1.02 | 0.61 | 0.52 |
|  | 15 years | 1568 | .76 | 0.00 | 6.00 | 1.76 | 1.09 | 0.71 | 0.36 |
|  | 18 years | 596 | .69 | 0.00 | 5.67 | 1.85 | 1.07 | 0.54 | -0.07 |
| Externalizing | 11 years | 2320 | .68 | 0.00 | 7.19 | 2.36 | 1.15 | 0.44 | 0.09 |
|  | 15 years | 1568 | .71 | 0.00 | 5.67 | 2.19 | 1.14 | 0.41 | -0.25 |
|  | 18 years | 596 | .70 | 0.00 | 5.67 | 2.06 | 1.12 | 0.41 | -0.18 |
| *Note*. Min. = minimum. Max. = maximum. *SD* = standard deviation. | | | | | | | | | |

| Table S2. Model fit of the latent growth models of the internalizing and externalizing symptoms on the total sample | | | | | | | | | |  |
| --- | --- | --- | --- | --- | --- | --- | --- | --- | --- | --- |
| Model | Estimated | Shape | χ^2^(df) | RMSEA (90% CI) | CFI | TLI | SRMR | AIC | BIC | |
| Parallel processes model | intercept, slope | linear | 25.396(4) | .046 (.030, .064) | .983 | .938 | .026 | 25710.443 | 25844.779 | |
| *Note.* RMSEA=root mean square of approximation; CFI=comparative fit index; TLI=Tucker-Lewis index; AIC=Akaike information criterion; BIC=Bayesian information criterion. | | | | | | | | | |  |

| Table S3. Model parameters and results of the 2-, 3-, 4-, and 5-class latent class growth models | | | | | | | | |
| --- | --- | --- | --- | --- | --- | --- | --- | --- |
| *N* of classes | Best Loglikelihood | AIC | BIC | Entropy | VLM RLR | LMR ALRT | BLRT | Smallest class % |
| 2 | -12904.174 | 25844.348 | 25949.480 | .610 | *p* < .001 | *p* < .001 | *p* < .001 | 15.03% |
| 3 | -12808.773 | 25663.545 | 25797.882 | .496 | *p* < .001 | *p* < .001 | *p* < .001 | 10.78% |
| 4 | -12778.192 | 25612.383 | 25775.923 | .517 | *p* = .026 | *p* = .029 | *p* < .001 | 7.87% |
| 5 | -12747.571 | 25561.143 | 25753.886 | .542 | *p* = .367 | *p* = .375 | *p* < .001 | 4.13% |
| *Note.* AIC=Akaike information criterion; BIC=Bayesian information criterion; VLM RLT=Vuong-Lo-Mendell-Rubin likelihood ratio test; LMR ALRT=Luo-Mendell-Rubin adjusted likelihood ratio test; BLRT=parametric bootstrap likelihood ratio test. | | | | | | | | |

| Table S4. Classification Probabilities for the Most Likely Latent Class Membership (Column) by Latent Class (Row) | | | | |
| --- | --- | --- | --- | --- |
|  | 1 | 2 | 3 | 4 |
| 1 | .892 | .024 | .064 | .020 |
| 2 | .301 | .497 | .198 | .005 |
| 3 | .393 | .062 | .493 | .053 |
| 4 | .255 | .003 | .128 | .613 |

| Table S5. Class proportions and mean intercept and slope results in the 4-class model | | | | | | |
| --- | --- | --- | --- | --- | --- | --- |
|  | *N* of class members | % of total *N* | Mean internalizing latent intercept factor | Mean internalizing latent slope factor | Mean externalizing latent intercept factor | Mean externalizing latent slope factor |
| Class O1 | 1629 | 64.08% | 1.714*** | -0.072*** | 1.945*** | -0.073*** |
| Class O2 | 200 | 7.87% | 1.639*** | -0.057*** | 3.625*** | -0.187*** |
| Class O3 | 482 | 18.96% | 2.148*** | 0.032+ | 2.699*** | 0.084** |
| Class O4 | 231 | 9.09% | 3.220*** | 0.017 | 2.517*** | -0.025 |
| *Note.* Variables in the analyses were divided by four at age 11 and by three at ages 15 and 18 to bring them into the same measurement scale (items were rated on a 0 to 3 scale at age 11 and on a 0 to 2 scale at ages 15 and 18). This means that the true intercepts and slopes performed on the original scores would be higher than the intercepts and slopes received in this analysis.  + *p* < .10. * *p* < .05. *** *p* < .001 | | | | | | |

| Table S6. Offspring mental health correlated with parental depressive symptoms at all available measurement points | | | | | | | | |
| --- | --- | --- | --- | --- | --- | --- | --- | --- |
|  | Father | | | | | | | |
|  | prenatal | newborn | 6 months | 18 months | 3 years | 5 years | 7 years | 11 years |
| SDQ: internalizing (11y) | .07*** | .06* | .08*** | .05 | .09*** | .08*** | .05* | .10*** |
| SDQ: externalizing (11y) | .02 | .05* | .06* | .04 | .06* | .04 | .01 | .06* |
| SDQ: internalizing (15y) | .07* | .05 | .07* | .06* | .05 | .07* | .06* | .09*** |
| SDQ: externalizing (15y) | .02 | .03 | .05 | .03 | <.01 | .05 | .07* | .07* |
| SDQ: internalizing (18y) | .12* | .16*** | .10* | .09* | .13*** | .09* | .11* | .13* |
| SDQ: externalizing (18y) | .01 | .09* | .05 | .04 | <.01 | .02 | -.02 | .05 |
| Self-esteem (19y) | -.06 | -.05 | -.06 | -.08 | .02 | .01 | -.11 | -.12 |
| Satisfaction with life (19y) | -.05 | <.01 | -.02 | -.03 | .06 | -.09 | -.16* | -.13 |
| Stressful life events (11y) | .02 | .04 | .04 | .08*** | .06*** | .05* | .06* | .04 |
| Stressful life events (15y) | .04 | .06* | .05 | .08*** | .03 | .07* | .07* | .09*** |
| Stressful life events (18y) | .04 | .08 | .13*** | .15*** | .08 | .12* | .10* | .18*** |
| Stressful life events (19y) | .09 | .09* | .12* | .17*** | .10* | .04 | .15*** | .15*** |
|  | Mother | | | | | | | |
|  | prenatal | newborn | 6 months | 18 months | 3 years | 5 years | 7 years | 11 years |
| SDQ: internalizing (11y) | .11*** | .11*** | .11*** | .11*** | .13*** | .12*** | .11*** | .09*** |
| SDQ: externalizing (11y) | .06* | .07*** | .09*** | .09*** | .10*** | .09*** | .09*** | .05* |
| SDQ: internalizing (15y) | .08*** | .07* | .07*** | .09*** | .09*** | .08*** | .13*** | .11*** |
| SDQ: externalizing (15y) | .12*** | .08*** | .10*** | .10*** | .11*** | .08*** | .10*** | .11*** |
| SDQ: internalizing (18y) | .11* | .05 | .04 | .03 | .07 | .05 | .10* | .09* |
| SDQ: externalizing (18y) | .09 | .12* | .09* | .06 | .06 | .06 | .08 | .06 |
| Self-esteem (19y) | -.04 | .02 | .02 | -.03 | .01 | .02 | -.02 | -.01 |
| Satisfaction with life (19y) | -.02 | .10 | .07 | -.03 | -.03 | -.05 | -.14* | -.05 |
| Stressful life events (11y) | .09*** | .09*** | .10*** | .11*** | .12*** | .10*** | .13*** | .11*** |
| Stressful life events (15y) | .10*** | .04 | .08*** | .12*** | .09*** | .07* | .15*** | .11*** |
| Stressful life events (18y) | .08 | .09* | .09* | .10* | .03 | .06 | .12*** | .07 |
| Stressful life events (19y) | .12* | .14*** | .11* | .20*** | .11* | .10* | .11* | .11* |
| *Note. M* = mean. *SD* = standard deviation. y = years of age. SDQ = Strength and Difficulties Questionnaire. The decision rule for including variables in this table was that there is at least one \|r\| > .10 in a row in either parent.  * *p* < .05. ** *p* < .01. *** *p* < .001. | | | | | | | | |

| Table S7. Multinomial regression predicting offspring internalizing and externalizing symptom trajectories (Classes O1-O4) with parental depressive symptom trajectories (Classes P1-P5) | | | |
| --- | --- | --- | --- |
|  | Class O2: Low internalizing and high externalizing symptoms | Class O3: Elevated internalizing and elevated externalizing symptoms | Class O4: High internalizing and elevated externalizing symptoms |
| Class P1: Mother has elevated depression, father is non-depressed | 1.43+ (0.98, 2.08) | 1.63*** (1.26, 2.11) | 1.70** (1.19, 2.42) |
| Class P2: Both mother and father have elevated depression | 1.45+ (0.97, 2.17) | 1.45* (1.09, 1.92) | 1.49* (1.01, 2.20) |
| Class P4: Both mother and father are constantly depressed | 1.59 (0.78, 3.23) | 1.18 (0.67, 2.06) | 2.59** (1.45, 4.65) |
| Class P5: Mother is constantly depressed, father has elevated depression | 2.04** (1.22, 3.43) | 2.46*** (1.73, 3.52) | 2.41*** (1.48, 3.90) |
| *Note.* The reference category in offspring symptom patterns is Class O1: Low internalizing and low externalizing symptoms, the reference category in parental symptom patterns is Class P3: Both mother and father are constantly non-depressed.  + *p* < .10. * *p* < .05. ** *p* < .01. *** *p* < .001. | | | |

| Table S8. Comparison of the four internalizing and externalizing symptoms trajectories across newborn temperament and emotional life | | | | | |  |
| --- | --- | --- | --- | --- | --- | --- |
|  | Class O1: Low internalizing and low externalizing symptoms | Class O2: Low internalizing and high externalizing symptoms | Class O3: Elevated internalizing and elevated externalizing symptoms | Class O4: High internalizing and elevated externalizing symptoms | *η*^2^ | |
| Newborn temperament |  |  |  |  |  | |
| Cranky (*M*, *SD*) | 3.21 (0.77) | 3.06 (0.81) | 3.22 (0.78) | 3.19 (0.82) | <.01+ | |
| Calm (*M*, *SD*) | 2.80 (0.91) | 2.81 (0.93) | 2.83 (0.90) | 2.88 (0.88) | <.01 | |
| Chatty (*M*, *SD*) | 1.78 (0.73) | 1.83 (0.79) | 1.80 (0.74) | 1.84 (0.78) | <.01 | |
| Whiny (*M*, *SD*) | 1.57 (0.66) | 1.58 (0.66) | 1.57 (0.60) | 1.58 (0.71) | <.01 | |
| Demanding (*M*, *SD*) | 2.44 (0.97) | 2.41 (0.95) | 2.54 (0.95) | 2.32 (0.98) | <.01* | |
| Angry (*M*, *SD*) | 1.52 (0.70) | 1.58 (0.73) | 1.56 (0.73) | 1.55 (0.73) | <.01 | |
| Clingy (*M*, *SD*) | 3.31 (0.66) | 3.34 (0.64) | 3.32 (0.65) | 3.27 (0.68) | <.01 | |
| Lively (*M*, *SD*) | 3.54 (0.58) | 3.52 (0.69) | 3.55 (0.57) | 3.49 (0.67) | <.01 | |
| Social (*M*, *SD*) | 3.15 (0.82) | 3.14 (0.89) | 3.20 (0.81) | 3.12 (0.90) | <.01 | |
| Closed (*M*, *SD*) | 1.38 (0.55) | 1.42 (0.57) | 1.40 (0.58) | 1.33 (0.53) | <.01 | |
| Stubborn (*M*, *SD*) | 1.59 (0.75) | 1.66 (0.75) | 1.66 (0.80) | 1.62 (0.81) | <.01 | |
| No interest (*M*, *SD*) | 1.31 (0.58) | 1.37 (0.61) | 1.28 (0.51) | 1.21 (0.52) | <.01* | |
| Satisfied (*M*, *SD*) | 3.41 (0.60) | 3.41 (0.61) | 3.42 (0.60) | 3.42 (0.59) | <.01 | |
| Bright (*M*, *SD*) | 3.39 (0.61) | 3.43 (0.67) | 3.41 (0.64) | 3.43 (0.64) | <.01 | |
| Emotional life |  |  |  |  |  | |
| Aggression (m.) (*M*, *SD*) | 7.27 (1.77) | 7.38 (1.83) | 7.73 (1.86) | 7.47 (1.88) | .01*** | |
| Aggression (f.) (*M*, *SD*) | 7.32 (1.86) | 7.47 (1.91) | 7.56 (2.04) | 7.37 (1.88) | <.01 | |
| Affection (m.) (*M*, *SD*) | 23.70 (3.57) | 23.59 (3.40) | 23.03 (3.69) | 24.01 (3.62) | .01** | |
| Affection (f.) (*M*, *SD*) | 23.91 (3.55) | 24.07 (3.75) | 23.41 (3.73) | 24.16 (3.47) | <.01* | |
| Love of the baby (*M*, *SD*) | 1.35 (0.57) | 1.46 (0.65) | 1.44 (0.71) | 1.40 (0.62) | <.01* | |
| *Note.* m = mother. f = father.  + *p* < .10. * *p* < .05. ** *p* < .01. *** *p* < .001. | | | | | |  |

| Table S9. Comparison of the four internalizing and externalizing symptoms trajectories across socioeconomic resources | | | | | |
| --- | --- | --- | --- | --- | --- |
|  | Class O1: Low internalizing and low externalizing symptoms | Class O2: Low internalizing and high externalizing symptoms | Class O3: Elevated internalizing and elevated externalizing symptoms | Class O4: High internalizing and elevated externalizing symptoms | *η*^2^/*V* |
| Socioeconomic resources |  |  |  |  |  |
| Deprivation (*M*, *SD*) | 7.82 (3.04) | 8.42 (3.21) | 8.27 (3.15) | 8.36 (3.21) | .01** |
| Financial help *n*, % | 306 (29.4%) | 45 (33.8%) | 87 (26.8%) | 49 (30.6%) | .04 |
| Income (*M*, *SD*) | 5734.41 (2796.11) | 5339.32 (1570.34) | 5599.8 (2559.16) | 5604.66 (2405.41) | <.01 |
| Living in own house *n*, % | 809 (62.2%) | 102 (61.8%) | 251 (64%) | 110 (57.3%) | .04 |
| Crowding (*M*, *SD*) | 1.57 (0.97) | 1.58 (0.88) | 1.58 (0.82) | 1.65 (1.21) | <.01 |
| Amenities (*M*, *SD*) | 5.79 (1.53) | 5.78 (1.56) | 5.64 (1.45) | 5.63 (1.48) | <.01 |
| Utilities (*M*, *SD*) | 2.93 (1.56) | 3.02 (1.51) | 2.99 (1.52) | 2.79 (1.71) | <.01 |
| Social network (m.) (*M*, *SD*) | 21.48 (3.91) | 21.27 (4.05) | 21.68 (3.73) | 21.20 (3.86) | <.01 |
| Social network (f.) (*M*, *SD*) | 21.57 (4.21) | 21.57 (4.02) | 21.74 (4.18) | 21.89 (3.85) | <.01 |
| Social support (m.) (*M*, *SD*) | 17.46 (3.98) | 17.26 (3.98) | 17.04 (3.99) | 17.24 (4.25) | <.01 |
| Social support (f.) (*M*, *SD*) | 19.20 (4.43) | 19.59 (4.40) | 19.07 (4.66) | 19.59 (4.35) | <.01 |
| Partner taking care of baby *n*, % | 1161 (77.7%) | 149 (79.3%) | 342 (76.7%) | 166 (79%) | .02 |
| Partner taking care (hw) (*M*, *SD*) | 16.95 (14.56) | 17.34 (14.89) | 16.71 (14.7) | 15.57 (13.6) | <.01 |
| Partner taking care (mth) (*M*, *SD*) | 1.22 (1.37) | 1.22 (1.32) | 1.27 (1.45) | 1.47 (1.61) | <.01 |
| Family taking care of baby *n*, % | 629 (42.4%) | 91 (50%) | 183 (41.4%) | 95 (45.2%) | .05 |
| Family taking care (hw) (*M*, *SD*) | 10.03 (11.96) | 10.68 (12.73) | 8.93 (10.95) | 9.80 (10.09) | <.01 |
| Family taking care (mth) (*M*, *SD*) | 1.70 (1.51) | 1.80 (1.55) | 1.96 (1.74) | 1.71 (1.56) | <.01 |
| Non-family taking care of baby *n*, % | 53 (3.6%) | 5 (2.8%) | 16 (3.7%) | 3 (1.4%) | .04 |
| Non-family taking care (hw) (*M*, *SD*) | 4.13 (4.77) | 8.40 (7.83) | 4.86 (6.22) | 2.50 (0.71) | .05 |
| Non-family taking care (mth) (*M*, *SD*) | 2.80 (2.02) | 3.60 (1.67) | 3.50 (1.98) | 2.50 (3.54) | .03 |
| *Note.* m = mother. f = father. hm = hours per week. mth = child’s age in months.  ** *p* < .01. | | | | | |

| Table S10. Comparison of the four internalizing and externalizing symptoms trajectories across parental history and parental depressive symptoms | | | | | |
| --- | --- | --- | --- | --- | --- |
|  | Class O1: Low internalizing and low externalizing symptoms | Class O2: Low internalizing and high externalizing symptoms | Class O3: Elevated internalizing and elevated externalizing symptoms | Class O4: High internalizing and elevated externalizing symptoms | *η*^2^ |
| Parental history |  |  |  |  |  |
| Stressful life events (m.) (*M*, *SD*) | 2.78 (2.63) | 3.13 (2.84) | 3.46 (2.71) | 3.63 (2.97) | .01*** |
| Stressful life events (f.) (*M*, *SD*) | 2.93 (2.42) | 3.03 (2.52) | 3.63 (2.84) | 3.57 (3.85) | .01*** |
| Parental care (m.) (*M*, *SD*) | 39.27 (5.98) | 39.17 (5.95) | 38.51 (6.39) | 38.95 (6.87) | <.01 |
| Parental care (f.) (*M*, *SD*) | 38.62 (5.23) | 38.45 (5.36) | 38.54 (5.76) | 38.64 (6.16) | <.01 |
| Overprotection (m.) (*M*, *SD*) | 22.13 (4.60) | 22.29 (5.05) | 22.68 (4.98) | 22.70 (4.66) | <.01 |
| Overprotection (f.) (*M*, *SD*) | 22.93 (4.74) | 22.78 (4.37) | 22.74 (4.89) | 22.68 (4.77) | <.01 |
| Home stability (m.) (*M*, *SD*) | 2.93 (0.48) | 2.89 (0.47) | 2.92 (0.46) | 2.96 (0.53) | <.01 |
| Home stability (f.) (*M*, *SD*) | 2.93 (0.54) | 2.97 (0.54) | 2.91 (0.55) | 2.92 (0.54) | <.01 |
| Sexual abuse (m.) (*M*, *SD*) | 0.37 (0.65) | 0.41 (0.67) | 0.44 (0.68) | 0.41 (0.67) | <.01 |
| Sexual abuse (f.) (*M*, *SD*) | 0.12 (0.40) | 0.18 (0.50) | 0.17 (0.50) | 0.13 (0.45) | <.01 |
| Parental depressive symptoms |  |  |  |  |  |
| Mother (prenatal) (*M*, *SD*) | 6.15 (4.36) | 6.23 (4.27) | 7.10 (4.62) | 6.96 (4.88) | .01** |
| Mother (newborn) (*M*, *SD*) | 6.32 (4.24) | 7.04 (4.73) | 7.20 (4.91) | 7.20 (4.67) | .01*** |
| Mother (6 months) (*M*, *SD*) | 5.77 (4.02) | 6.54 (4.40) | 6.57 (4.55) | 6.62 (4.31) | .01*** |
| Mother (18 months) (*M*, *SD*) | 5.70 (4.11) | 6.50 (4.63) | 6.79 (4.53) | 6.47 (4.69) | .01*** |
| Mother (3 years) (*M*, *SD*) | 6.06 (4.38) | 6.45 (4.71) | 7.24 (4.73) | 7.24 (4.88) | .01*** |
| Mother (5 years) (*M*, *SD*) | 5.78 (4.48) | 6.30 (4.76) | 6.72 (4.81) | 6.67 (4.88) | .01*** |
| Mother (7 years) (*M*, *SD*) | 6.01 (4.55) | 6.45 (4.48) | 7.02 (4.87) | 7.34 (5.58) | .01*** |
| Mother (11 years) (*M*, *SD*) | 7.85 (4.68) | 8.01 (4.56) | 8.63 (4.88) | 8.81 (5.58) | .01** |
| Father (prenatal) (*M*, *SD*) | 4.24 (3.64) | 4.18 (3.55) | 4.31 (3.61) | 4.78 (4.13) | <.01 |
| Father (newborn) (*M*, *SD*) | 3.90 (3.45) | 4.06 (3.23) | 4.04 (3.23) | 4.25 (3.67) | <.01 |
| Father (6 months) (*M*, *SD*) | 3.73 (3.42) | 4.10 (3.25) | 3.98 (3.41) | 4.26 (3.70) | <.01+ |
| Father (18 months) (*M*, *SD*) | 4.10 (3.52) | 4.43 (3.69) | 4.34 (3.96) | 4.69 (4.55) | <.01 |
| Father (3 years) (*M*, *SD*) | 4.12 (3.64) | 4.05 (3.24) | 4.26 (3.71) | 4.60 (4.10) | <.01 |
| Father (5 years) (*M*, *SD*) | 4.04 (3.88) | 4.23 (3.40) | 4.32 (4.02) | 4.73 (4.60) | <.01 |
| Father (7 years) (*M*, *SD*) | 4.26 (4.00) | 4.39 (3.62) | 4.18 (3.81) | 4.76 (4.14) | <.01 |
| Father (11 years) (*M*, *SD*) | 6.13 (3.88) | 6.23 (4.19) | 6.43 (4.14) | 7.28 (4.11) | .01** |
| *Note.* m = mother. f = father. y = years of age.  + *p* < .10. ** *p* < .01. *** *p* < .001. | | | | | |

| Table S11. Multinomial regression predicting offspring internalizing and externalizing symptom trajectories (Classes O1-O4) with parental depressive symptom trajectories (Classes P1-P5) controlled for offspring sex and parental prenatal age on a subset of offspring who all have class membership probabilities ≥ 50% (*n* = 1708) | | | |
| --- | --- | --- | --- |
|  | Class O2: Low internalizing and high externalizing symptoms | Class O3: Elevated internalizing and elevated externalizing symptoms | Class O4: High internalizing and elevated externalizing symptoms |
| Offspring sex (female) | 0.65* (0.44, 0.97) | 1.39* (1.06, 1.82) | 1.64** (1.17, 2.31) |
| Mother’s age | 1.00 (0.94, 1.06) | 1.03 (0.99, 1.07) | 1.01 (0.96, 1.06) |
| Father’s age | 0.99 (0.95, 1.04) | 0.99 (0.96, 1.02) | 1.01 (0.97, 1.05) |
| Class P1: Mother has elevated depression, father is non-depressed | 1.87** (1.17, 2.98) | 1.41* (1.01, 1.97) | 1.73* (1.12, 2.66) |
| Class P2: Both mother and father have elevated depression | 1.54 (0.91, 2.63) | 1.20 (0.83, 1.75) | 1.85** (1.18, 2.92) |
| Class P4: Both mother and father are constantly depressed | 0.97 (0.29, 3.29) | 1.49 (0.75, 2.93) | 2.77** (1.33, 5.75) |
| Class P5: Mother is constantly depressed, father has elevated depression | 1.73 (0.80, 3.72) | 2.42*** (1.53, 3.84) | 2.16* (1.15, 4.05) |
| *Note.* The reference category in offspring symptom patterns is Class O1: Low internalizing and low externalizing symptoms, the reference category in parental symptom patterns is Class P3: Both mother and father are constantly non-depressed. Results are presented in odds-ratios.  + *p* < .10. * *p* < .05. ** *p* < .01. *** *p* < .001. | | | |

| Table S12. Comparison of the four internalizing and externalizing symptoms trajectories across parental relationship maintenance, demographic data, and offspring mental health on a subset of offspring who all have class membership probabilities ≥ 50% (*n* = 2128) | | | | | |
| --- | --- | --- | --- | --- | --- |
|  | Class O1: Low internalizing and low externalizing symptoms | Class O2: Low internalizing and high externalizing symptoms | Class O3: Elevated internalizing and elevated externalizing symptoms | Class O4: High internalizing and elevated externalizing symptoms | *η*^2^/*V* |
| Parental relationship maintenance |  |  |  |  |  |
| Unstable relationship *n*, % | 242 (16.6%) | 27 (19.0%) | 65 (19.8%) | 32 (16.5%) | .03 |
| Continuously married *n*, % | 1017 (69.5%) | 92 (64.8%) | 206 (62.6%) | 122 (62.9%) | .06* |
| Only married *n*, % | 1023 (69.9%) | 92 (64.8%) | 206 (62.6%) | 124 (63.9%) | .07* |
| Married ever *n*, % | 1394 (95.3%) | 131 (92.3%) | 304 (92.4%) | 182 (93.8%) | .05+ |
| Not first marriage *n*, % | 238 (16.3%) | 27 (19.0%) | 59 (17.9%) | 41 (21.1%) | .04 |
| Married later *n*, % | 80 (5.6%) | 8 (5.9%) | 15 (4.7%) | 11 (5.7%) | .01 |
| Bereavement *n*, % | 34 (2.3%) | 7 (4.9%) | 13 (4.0%) | 3 (1.5%) | .06+ |
| Separated *n*, % | 73 (5.0%) | 7 (4.9%) | 26 (7.9%) | 8 (4.1%) | .05 |
| Divorced *n*, % | 138 (9.4%) | 23 (16.2%) | 49 (14.9%) | 24 (12.4%) | .08** |
| Single ever *n*, % | 102 (7.0%) | 8 (5.6%) | 26 (7.9%) | 21 (10.8%) | .05 |
| Continuous cohabitation *n*, % | 1340 (91.6%) | 124 (87.3%) | 290 (88.1%) | 177 (91.2%) | .05 |
| Demographic data |  |  |  |  |  |
| Child’s sex (female) *n*, % | 721 (49.3%) | 54 (38.0%) | 192 (58.4%) | 116 (59.8%) | .11*** |
| Town (Brno) *n*, % | 1226 (83.8%) | 129 (90.8%) | 329 (100.0%) | 175 (90.2%) | .18*** |
| Age (m.) (*M*, *SD*) | 25.09 (4.68) | 25.01 (4.48) | 25.59 (5.23) | 25.43 (4.98) | <.01 |
| Age (f.) (*M*, *SD*) | 28.15 (5.72) | 27.98 (6.10) | 28.42 (6.39) | 28.73 (6.55) | <.01 |
| Education (m.) (*M*, *SD*) | 4.23 (1.98) | 4.27 (1.95) | 4.46 (1.96) | 4.23 (1.80) | <.01 |
| Education (f.) (*M*, *SD*) | 4.24 (2.24) | 3.97 (2.12) | 4.34 (2.23) | 4.11 (2.14) | <.01 |
| Employed (f.) *n*, % | 1386 (93.9%) | 171 (95.5%) | 395 (91.6%) | 189 (93.1%) | .02 |
| Offspring mental health |  |  |  |  |  |
| Stressful life events (11y) (*M*, *SD*) | 0.39 (0.33) | 0.50 (0.40) | 0.53 (0.39) | 0.61 (0.40) | .05*** |
| Stressful life events (15y) (*M*, *SD*) | 0.33 (0.26) | 0.41 (0.33) | 0.62 (0.41) | 0.57 (0.37) | .14*** |
| Stressful life events (18y) (*M*, *SD*) | 0.38 (0.24) | 0.47 (0.28) | 0.60 (0.31) | 0.53 (0.26) | .12*** |
| Stressful life events (19y) (*M*, *SD*) | 0.28 (0.23) | 0.39 (0.31) | 0.43 (0.34) | 0.46 (0.39) | .07*** |
| Self-esteem (15y) (*M*, *SD*) | 30.70 (4.01) | 30.66 (4.46) | 27.24 (4.22) | 26.62 (4.95) | .14*** |
| Self-esteem (18y) (*M*, *SD*) | 32.06 (4.32) | 30.73 (3.81) | 28.08 (4.69) | 27.25 (5.58) | .17*** |
| Self-esteem (19y) (*M*, *SD*) | 32.41 (5.17) | 33.00 (3.74) | 28.95 (5.80) | 26.93 (5.08) | .14*** |
| Satisfaction with life (15y) (*M*, *SD*) | 18.41 (3.69) | 17.95 (3.64) | 16.12 (4.02) | 15.83 (4.25) | .08*** |
| Satisfaction with life (19y) (*M*, *SD*) | 18.56 (4.29) | 17.11 (4.26) | 16.54 (4.61) | 15.17 (4.76) | .07** |
| *Note.* m = mother. f = father. y = years of age.  * *p* < .05. ** *p* < .01. *** *p* < .001. | | | | | |

| Table S13. Comparison of the four internalizing and externalizing symptoms trajectories across obstetric history and parental health on a subset of offspring who all have class membership probabilities ≥ 50% (*n* = 2128) | | | | | |
| --- | --- | --- | --- | --- | --- |
|  | Class O1: Low internalizing and low externalizing symptoms | Class O2: Low internalizing and high externalizing symptoms | Class O3: Elevated internalizing and elevated externalizing symptoms | Class O4: High internalizing and elevated externalizing symptoms | *η*^2^/*V* |
| Obstetric history |  |  |  |  |  |
| Previous pregnancy *n*, % | 736 (62.8%) | 75 (65.2%) | 176 (64.2%) | 99 (63.1%) | .02 |
| First pregnancy with partner *n*, % | 142 (19.7%) | 18 (24.7%) | 44 (25.7%) | 21 (21.6%) | .06 |
| Miscarriage *n*, % | 176 (23.9%) | 21 (28.4%) | 42 (24.0%) | 34 (34.3%) | .07 |
| Abortion *n*, % | 237 (32.2%) | 30 (40.0%) | 81 (46.0%) | 32 (32.7%) | .11** |
| Birth of dead child ever *n*, % | 27 (3.7%) | 4 (5.4%) | 8 (4.5%) | 6 (6.1%) | .04 |
| Obs. treatment *n*, % | 40 (36.0%) | 6 (46.2%) | 12 (35.3%) | 11 (42.3%) | .07 |
| *N* of previous pregnancies (*M*, *SD*) | 1.73 (1.05) | 2.01 (1.60) | 2.00 (1.17) | 1.93 (1.44) | .01+ |
| *N* of children (*M*, *SD*) | 1.01 (0.68) | 1.01 (0.68) | 1.06 (0.74) | 0.98 (0.74) | <.01 |
| *N* of miscarriage (*M*, *SD*) | 1.16 (0.50) | 1.20 (0.41) | 1.19 (0.45) | 1.27 (0.67) | .01 |
| *N* of abortion (*M*, *SD*) | 1.31 (0.60) | 1.40 (0.77) | 1.38 (0.64) | 1.44 (0.91) | <.01 |
| Parental health |  |  |  |  |  |
| Smoking (m.) *n*, % | 152 (12.0%) | 21 (16.5%) | 41 (14.1%) | 18 (10.6%) | .04 |
| Smoking (f.) *n*, % | 410 (36.3%) | 42 (37.8%) | 113 (42.5%) | 67 (42.7%) | .06 |
| Substance use (m.) *n*, % | 16 (1.4%) | 1 (0.9%) | 4 (1.5%) | 5 (3.2%) | .04 |
| Substance use (f.) *n*, % | 39 (2.7%) | 6 (4.4%) | 11 (3.5%) | 8 (4.3%) | .03 |
| *N* of diseases (m.) (*M*, *SD*) | 0.68 (1.00) | 0.66 (1.04) | 0.98 (1.19) | 0.93 (1.19) | .01*** |
| *N* of diseases (f.) (*M*, *SD*) | 0.71 (1.06) | 0.78 (1.26) | 0.79 (1.36) | 0.85 (1.17) | <.01 |
| Alcohol use (m.) (*M*, *SD*) | 0.14 (0.64) | 0.16 (0.60) | 0.11 (0.53) | 0.21 (0.79) | <.01 |
| Alcohol use (f.) (*M*, *SD*) | 1.60 (1.32) | 1.66 (1.40) | 1.70 (1.39) | 1.60 (1.40) | <.01 |
| Psychotropic pills (m.) (*M*, *SD*) | 1.04 (0.22) | 1.09 (0.36) | 1.07 (0.33) | 1.10 (0.36) | .01* |
| Psychotropic pills (f.) (*M*, *SD*) | 1.04 (0.25) | 1.06 (0.26) | 1.04 (0.19) | 1.08 (0.38) | <.01 |
| *Note.* m = mother. f = father.  + *p* < .10. * *p* < .05. ** *p* < .01. *** *p* < .001. | | | | | |

| **a**  **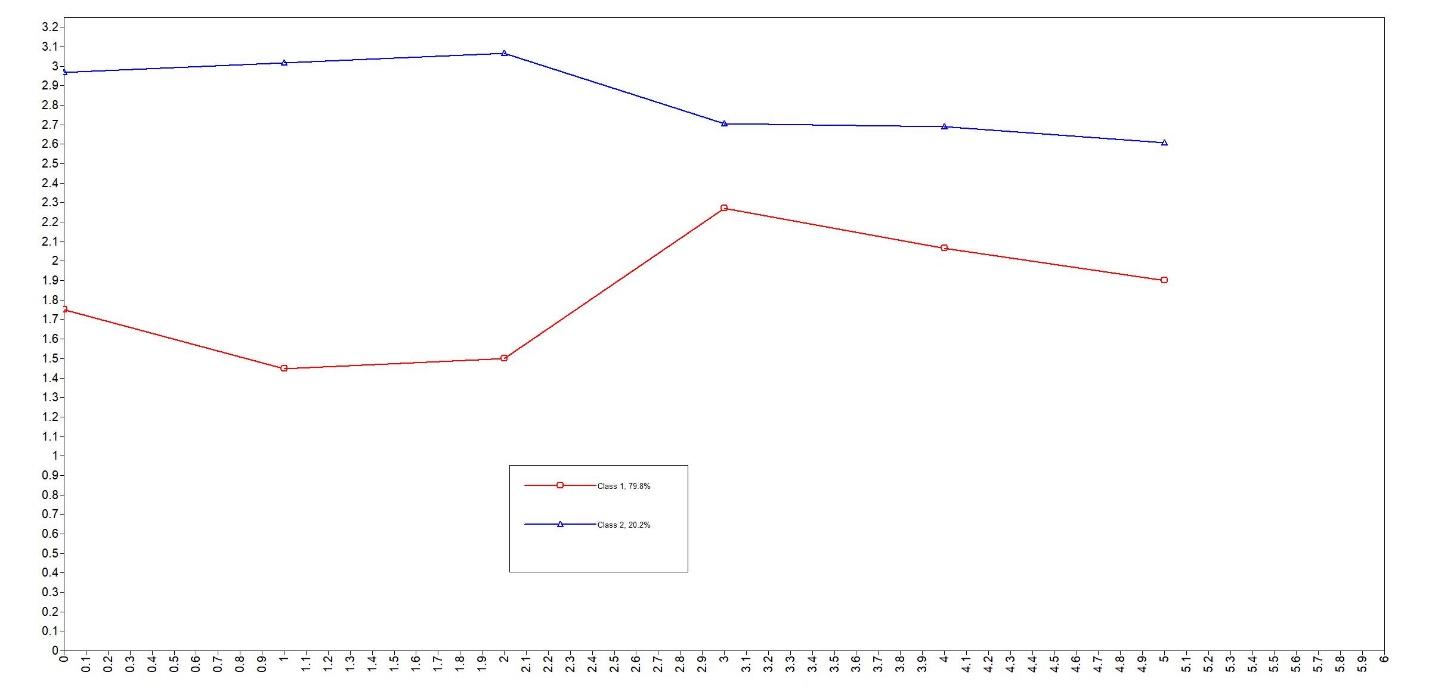** |
| --- |
| **b**  **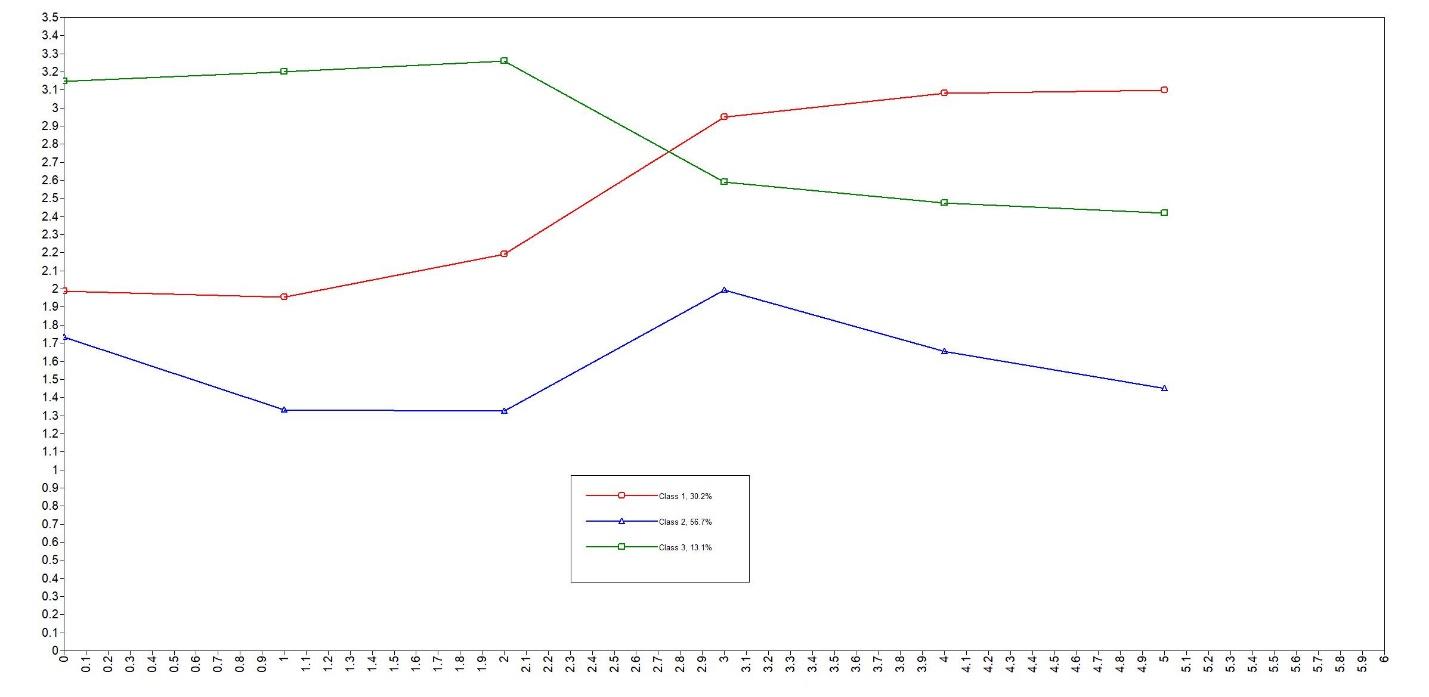** |
| **c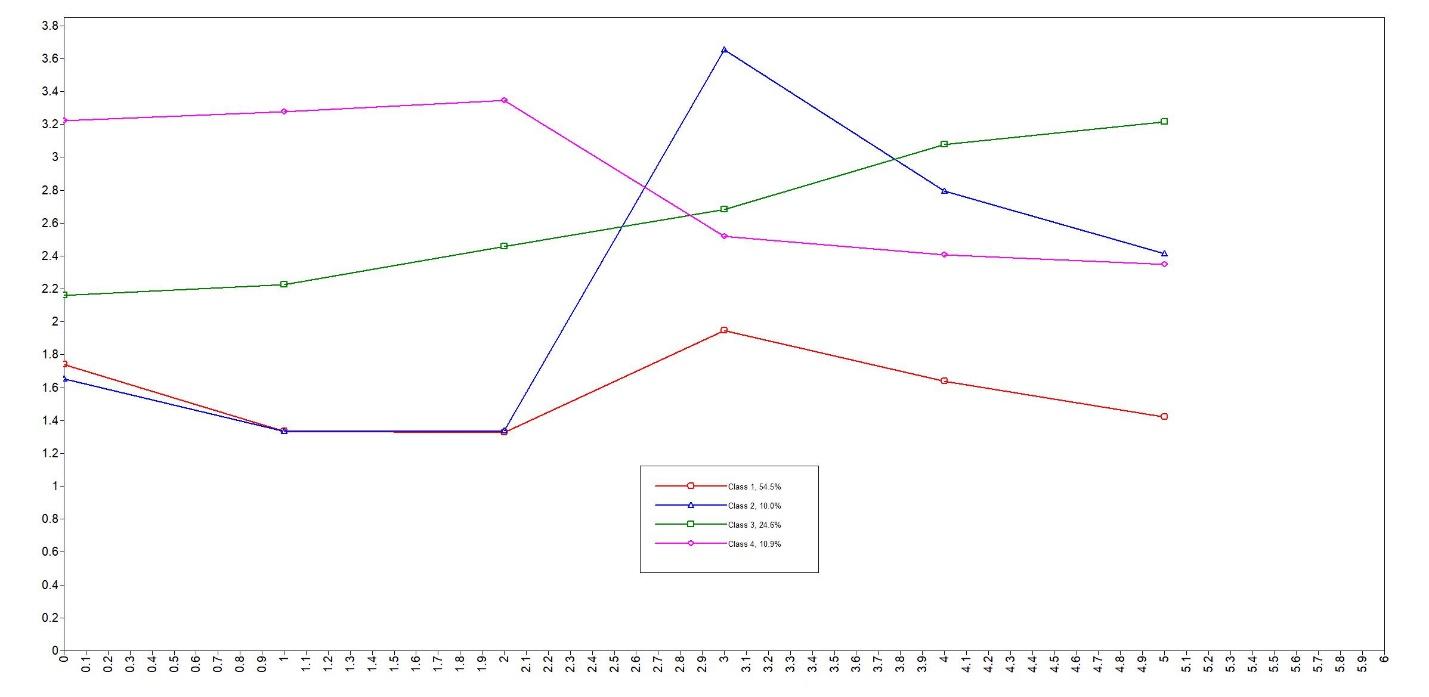** |
| **d**  **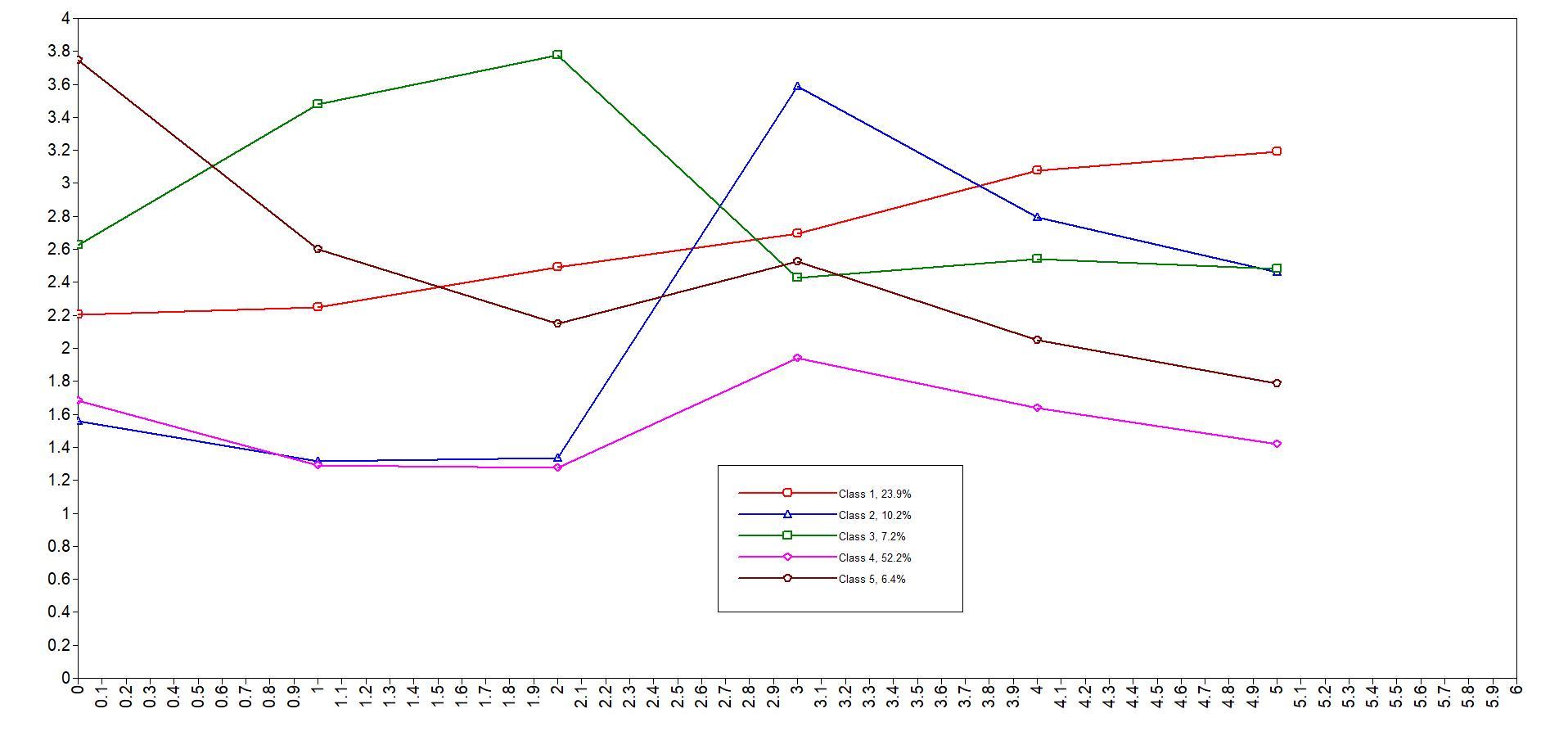** |
| **Figure S1.** Mean internalizing and externalizing symptoms (left: 0, 1, and 2 points on the x axis; and right: 3, 4, and 5 points on the x axis within each diagram, respectively) in each model extracting 2 to 5 classes. **a**: 2-class model; **b**: 3-class model; **c**: 4-class model; **d**: 5-class model. |

| **a**  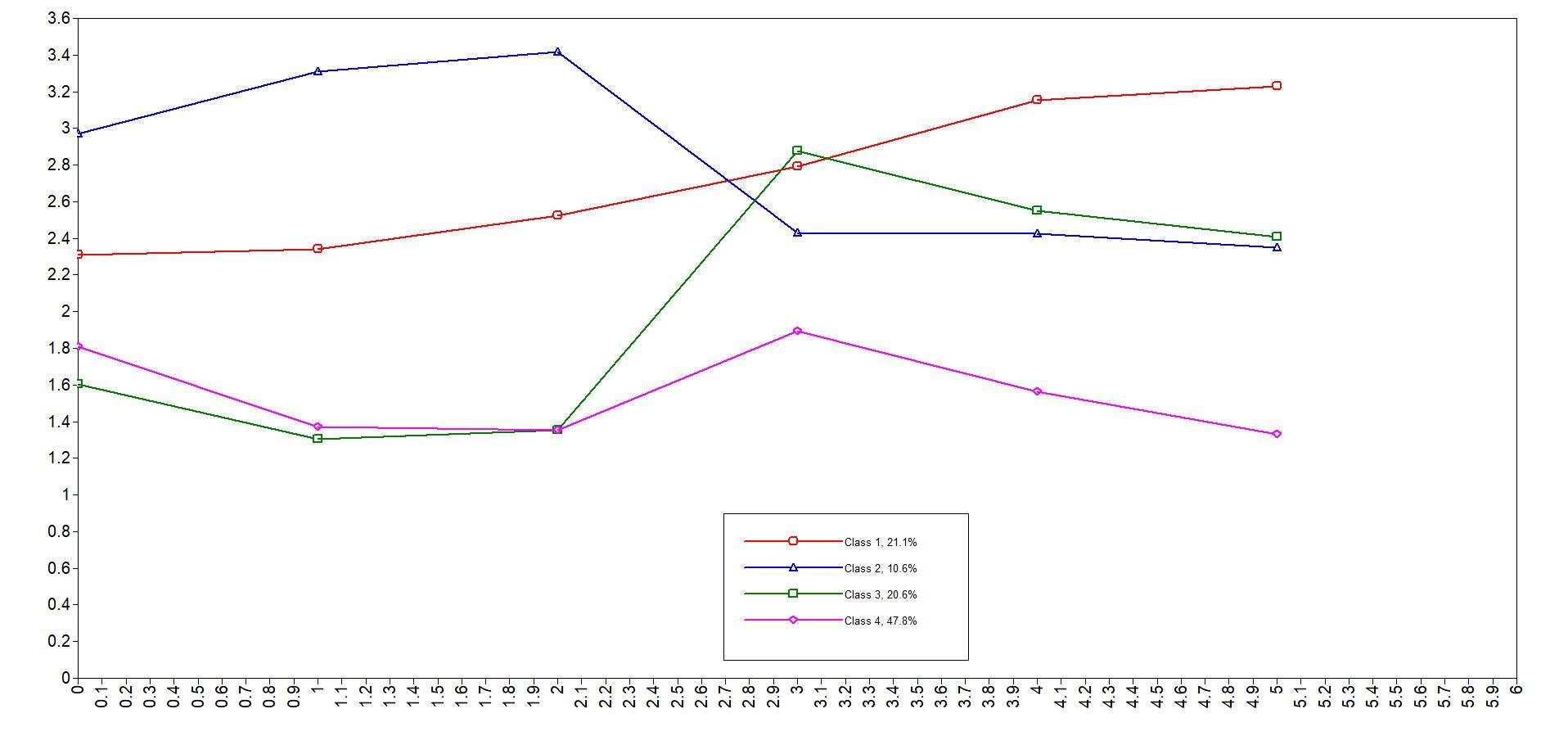 |
| --- |
| **b**  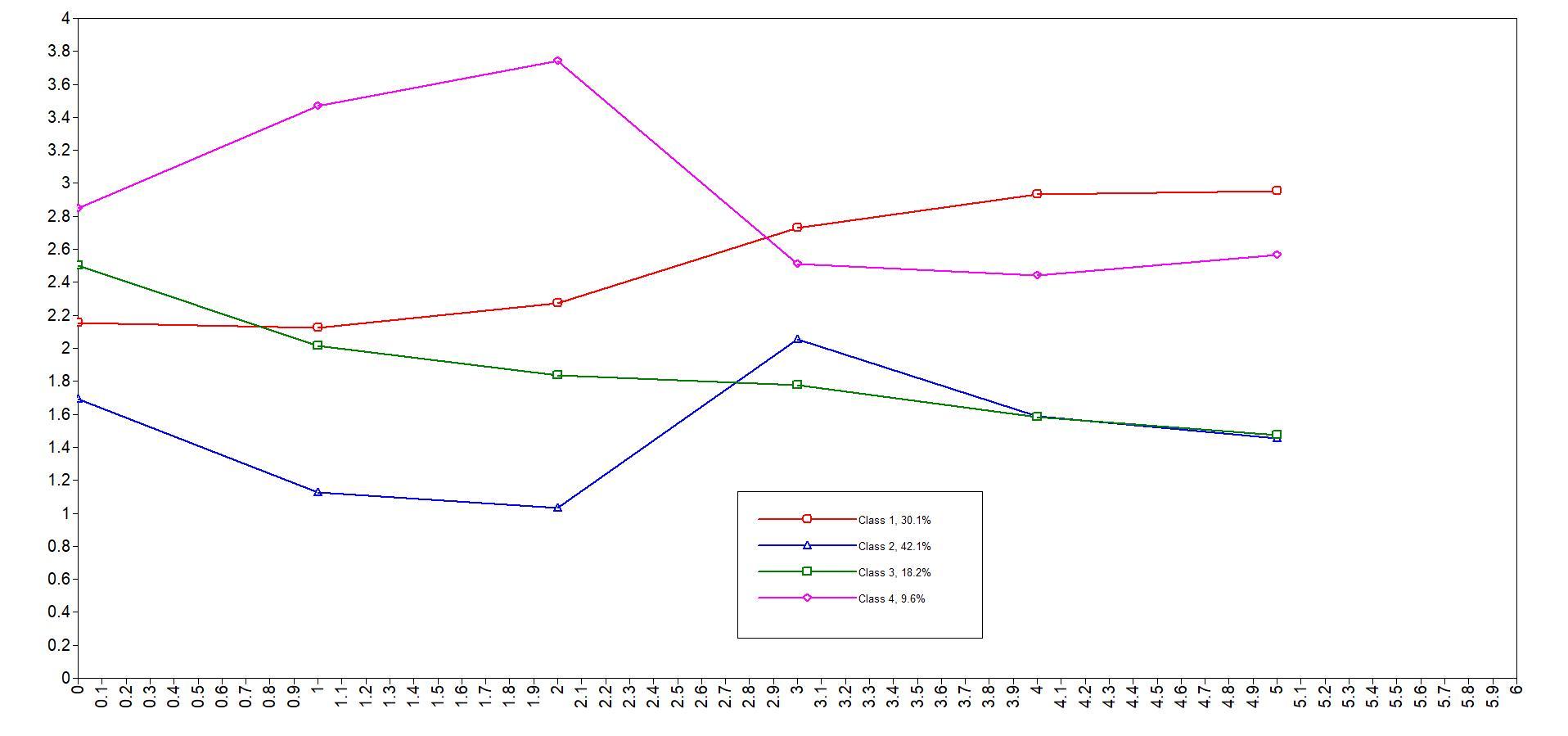 |
| **Figure S2.** Patterns yielded from the sensitivity analysis obtained on subsets of the sample with varying data coverage. Mean internalizing and externalizing symptoms (left: 0, 1, and 2 points on the x axis; and right: 3, 4, and 5 points on the x axis within each diagram, respectively) in each model extracting 4 classes. **a**: good data coverage (both internalizing and externalizing symptoms available for at least 2 time points out of 3, *n* = 1459); **b**: full data coverage (both internalizing and externalizing symptoms available for all 3 measurement points, *n* = 469). |

**References**

Akaike, H. (1973). Information theory as an extension of the maximum likelihood principle. In B. N. Petrov & F. Csaki (Eds.), *Second international symposium on information theory* (p. 267). Budapest, Hungary: Akademiai Kiado.

Brown, T. A. (2006). *Confirmatory factory analysis for applied research.* Guilford Press.

Lo, Y., Mendell, N., & Rubin, D. (2001). Testing the number of components in a normal mixture. *Biometrika, 88*, 767–778.

McLachlan, G., & Peel, D. (2004). *Finite mixture models*. New York, NY: Wiley.

Schwarz, G. (1978). Estimating the dimension of a model. *The Annals of Statistics, 6,* 461–464.

Van De Schoot, R., Sijbrandij, M., Winter, S. D., Depaoli, S., & Vermunt, J. K. (2017). The GRoLTS-checklist: guidelines for reporting on latent trajectory studies. *Structural Equation Modeling: A Multidisciplinary Journal*, *24*, 451-467.

Vuong, Q. (1989). Likelihood ratio tests for model selection and non-nested hypotheses. *Econometrica, 57*, 307-333.
